# Epigenetic Regulations of Perineural Invasion in Head and Neck Squamous Cell Carcinoma

**DOI:** 10.3389/fgene.2022.848557

**Published:** 2022-04-27

**Authors:** Pavel Hurník, Zuzana Chyra, Tereza Ševčíková, Jan Štembírek, Kateřina Smešný Trtková, Daria A. Gaykalova, Marcela Buchtová, Eva Hrubá

**Affiliations:** ^1^ Department of Clinical and Molecular Pathology and Medical Genetics, Faculty of Medicine and University Hospital Ostrava, Ostrava, Czechia; ^2^ Department of Histology and Embryology, Medical Faculty, Masaryk University, Brno, Czechia; ^3^ Department of Hematooncology, University Hospital Ostrava, Ostrava, Czechia; ^4^ Department of Maxillofacial Surgery, University Hospital Ostrava, Ostrava, Czechia; ^5^ Laboratory of Molecular Morphogenesis, Institute of Animal Physiology and Genetics, Czech Academy of Sciences, Brno, Czechia; ^6^ Department of Clinical and Molecular Pathology, Faculty of Medicine and University Hospital Olomouc, Olomouc, Czechia; ^7^ Department of Otorhinolaryngology-Head and Neck Surgery, University of Maryland Medical Center, Baltimore, MD, United States; ^8^ Marlene and Stewart Greenebaum Comprehensive Cancer Center, University of Maryland Medical Center, Baltimore, MD, United States; ^9^ Institute for Genome Sciences, University of Maryland Medical Center, Baltimore, MD, United States; ^10^ Department of Oncology, Sidney Kimmel Comprehensive Cancer Center, Johns Hopkins University, Baltimore, MD, United States; ^11^ Department of Experimental Biology, Faculty of Science, Masaryk University, Brno, Czechia

**Keywords:** HNSCC, perineural invasion, epigenetics, oral cancer, miRNAs

## Abstract

Carcinomas of the oral cavity and oropharynx belong among the ten most common malignancies in the human population. The prognosis of head and neck squamous cell carcinoma (HNSCC) is determined by the degree of invasiveness of the primary tumor and by the extent of metastatic spread into regional and distant lymph nodes. Moreover, the level of the perineural invasion itself associates with tumor localization, invasion’s extent, and the presence of nodal metastases. Here, we summarize the current knowledge about different aspects of epigenetic changes, which can be associated with HNSCC while focusing on perineural invasion (PNI). We review epigenetic modifications of the genes involved in the PNI process in HNSCC from the omics perspective and specific epigenetic modifications in OSCC or other neurotropic cancers associated with perineural invasion. Moreover, we summarize DNA methylation status of tumor-suppressor genes, methylation and demethylation enzymes and histone post-translational modifications associated with PNI. The influence of other epigenetic factors on the HNSCC incidence and perineural invasion such as tobacco, alcohol and oral microbiome is overviewed and HPV infection is discussed as an epigenetic factor associated with OSCC and related perineural invasion. Understanding epigenetic regulations of axon growth that lead to tumorous spread or uncovering the molecular control of axon interaction with cancer tissue can help to discover new therapeutic targets for these tumors.

## 1 Introduction

Squamous cell carcinoma of the head and neck (HNSCC) originates from the mucosal lining of the upper aerodigestive tract, thus including cancers of the oral cavity, paranasal sinuses, nasal cavity, pharynx, and larynx. According to recent estimates, more than 650,000 new cases are diagnosed, and over 350,000 cancer deaths are reported every year worldwide (Torre et al., 2012). From these tumors, oral squamous cell carcinoma (OSCC) represents the most common oral malignancy, demonstrating up to 80–90% of all malignant neoplasms of the oral cavity (Johnson et al., 2000). Despite supporting health education and improving awareness among the general public, and primary care practitioners, many patients are diagnosed in advanced stages of disease (stages III and IV). In addition, the etiology of SCC (squamous cell carcinoma) is typically linked to tobacco and alcohol abuse (Mello et al., 2019). However, emerging evidence revealed an increasing proportion of oropharyngeal tumors caused by human papillomavirus (HPV) infection (Miller and Johnstone, 2001). Therefore, two biologically distinct types of HNSCC (HPV-positive and HPV-negative) can be distinguished. In the United States, HPV-positive oropharyngeal cancer is the fastest rising malignant disease in young white men (Gillison et al., 2019). This alarming trend is expected to expand soon into other economically developed countries (Kreimer et al., 2020).

Several biological and molecular criteria have been established to determine the histopathological staging of SCC, which play a key role in post-surgery treatment and estimation of prognosis of oral and oropharyngeal carcinomas (Woolgar, 2006). Staging and grading are based on the localization and volume of tumor, histopathological grading, histological type of squamous cell carcinoma, positivity of edges, the incidence of regional and distant metastasis, and the other signs of aggressive behavior (perineural invasion, endovascular invasion, etc.). Because the degree of primary tumor invasiveness and extent of metastatic spread into regional and distant lymph nodes closely correlate with the level of PNI, we will focus on PNI in HNSCC and epigenetic regulation of genes contributing to the cancer progression.

## 2 SCC and Perineural Invasion

**FIGURE 1:**
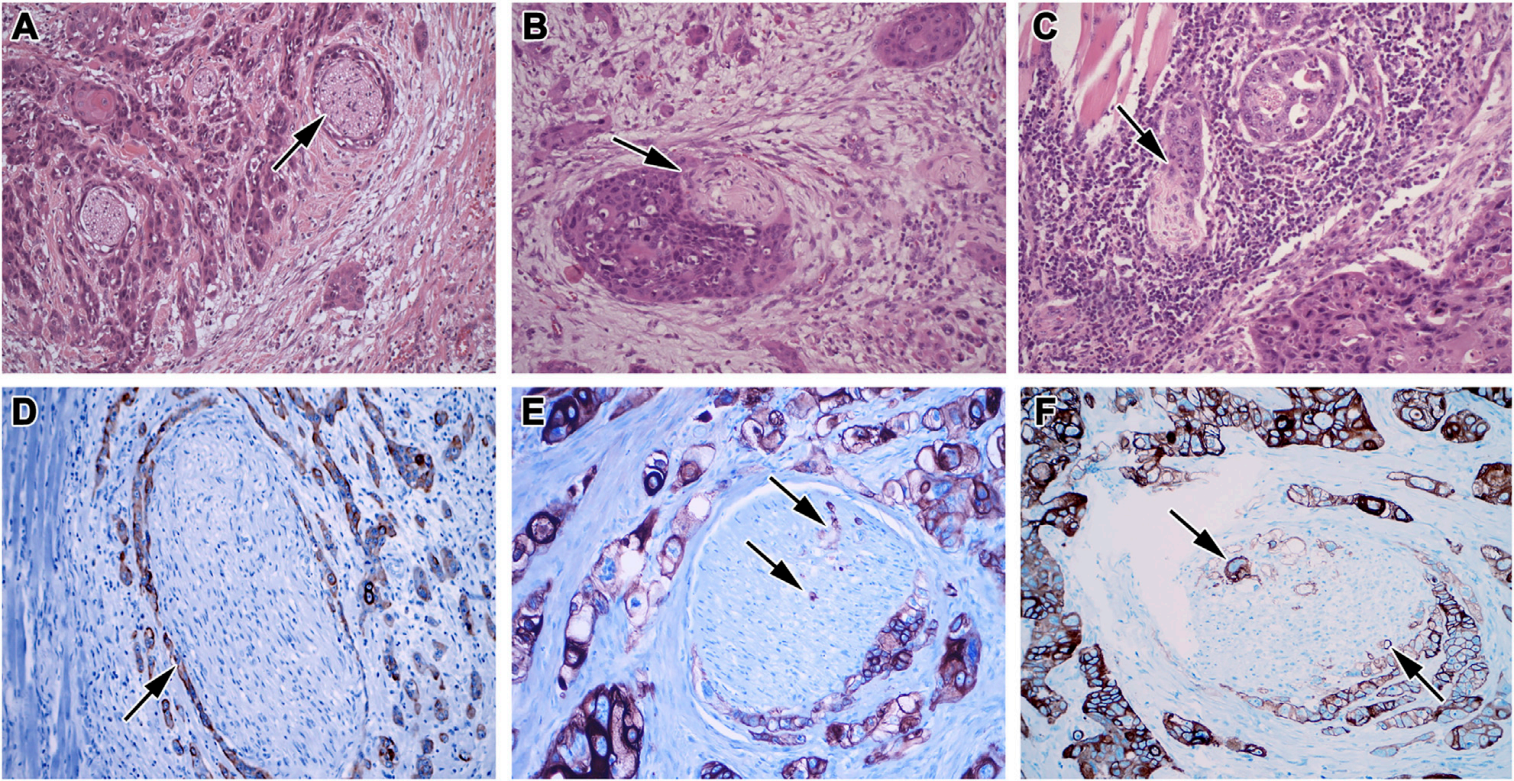
Morphology of perineural invasion. **(A)** Complete circular PNI in the invasive front of the tumor, **(B)** Partial PNI in more than 30% of the nerve, **(C)** Partial PNI in less than 30% of the nerve, HE, 200x. **(D)** Complete circular PNI without intraneural propagation, **(E)** Partial circular PNI in less than 50% of the nerve with focal intraneural propagation, **(F)** Partial circular PNI in more than 50% of the nerve with focal intraneural propagation, immunohistochemistry, cytokeratin AE1/A3 (DAB, brown), nuclei (HE, blue), 200x.

Perineural tumor spreading is defined as the ability of tumor cells to penetrate into, around, or through the nerve tissue (Chatzistefanou et al., 2017) (Figure 1). Recently, this process has been described in colorectal carcinoma and salivary gland malignancies (Liebig et al., 2009a; Speight and Barett, 2009b). A possible cause of the perineural spread may lie in the chemotropism of tumor cells that can be stimulated by nerve tissue to grow further. Such interactions also act reciprocally, and cancer cells can induce the growth of the neural tissue. This process increases the invasion of the tumor into surrounding tissue and causes cancer to spread into areas located relatively distant from the primary tumor site.

Primary cell types involved in PNI are neural Schwann cells (SCs) (Deborde and Wong, 2017), and cancer-associated fibroblasts (Zhang et al., 2019). OSCC is a neurotropic malignancy, and SCs are key mediators and promoters of PNI (Azam and Pecot, 2016; Deborde and Wong, 2017). During interaction with tumorous tissue, their activity recapitulates the normal Schwann cells’ response to peripheral nerve injury, i.e., dynamic transcriptional reprogramming of these cells to active dedifferentiated subtype. It is interesting to note that such reprogramming requires epigenomic regulation through modification of chromatin structure (Ma et al., 2016).

Previously, the invasion was thought to be caused by a mechanical incursion of the tumorous tissue through the relatively thin epineurium tissue (Bockman et al., 1994). However, this concept was rejected after the improved microscopic techniques demonstrated that the tumor cells did not grow passively around the nerves but instead penetrated the perineurium close to Schwann cells and axons up to endoneurium (Gil et al., 2009). Rarely, some less aggressive tumors also develop this nerve-tumor complex at relatively early stages, while others, more aggressive tumors, cause PNI at advanced stages (Chen et al., 2007). One possible explanation is the formation of specific microenvironments in the perineural space, which may contain cellular factors that act on neural tissue and certain types of tumorous tissues. cDNA microarray analysis of adenoid cystic carcinoma samples with and without perineural invasion, deregulation of genes controlling cell cycle, cytoskeleton, and extracellular matrix has been demonstrated. The extracellular matrix also contained increased amounts of neurotrophic factors and adhesive molecules, promoting tumor spreading based on chemotaxis (Liebig et al., 2009b). However, similar analyses have not been performed for HNSCC in relation to PNI yet.

Previously, it was proposed to define PNI as a tumor invasion into the proximity of the nerve comprising 1/3 of the nerve circuit and/or displaying the presence of cancer cells in any of three nerve layers (Fagan et al., 1998). Unfortunately, this classification cannot differentiate between perineural proliferation without infiltration of the nerve fascicle and intraneural propagation or penetration of the cells directly into the nerve, which may reflect a direct clinical impact and affect the patient’s prognosis. This fact also illustrates the need for a more comprehensive examination of regulatory processes, which can potentially lead to distinguishing between these two behaviors and serve as an essential indicator of the tumor aggressiveness, thus giving information about survival prediction and/or probability of the local tumor recurrence.

In clinical settings, the occurrence of perineural invasion among cancer patients usually ranges from 2–30% but can reach up to 82%, depending on the cancer type and the diagnostic methods (O’Brien et al., 1986; Soo et al., 1986; Kurtz et al., 2005). Perineural invasion of squamous cell carcinoma has been reported, but not fully investigated. In clinical trials investigating SCC in the orofacial area, this invasion can be a significant independent factor associated with increased local recurrence of the tumor, metastases, and median patient survival (Sutton et al., 2003; Binmadi and Basile, 2011; Jardim et al., 2015). In the orofacial area, perineural invasion can cause intracranial spreading of the lip carcinoma along the facial or trigeminal nerve branch, thus negatively affecting the patient survival rate (Caldemeyer et al., 1998; Sullivan and Smee, 2006). Previous findings are, however, not consistent as some studies have not found a statistically significant correlation between lymphogenic metastases and perineural invasion and carcinomas in the oral cavity (Wallwork et al., 2007), while other sources confirm this correlation as statistically significant (Woolgar and Scott, 1995; Rahima et al., 2004; Woolgar, 2006). Nevertheless, a relationship was found between the lymphogenic and perineural invasion of T1-T2 carcinomas in the anterior part of the tongue (Larsen et al., 2009). Also, tumor thickness, “non-cohesive invasion front,” neural and bone invasion were described as powerful factors affecting the lymphogenic spreading of the tumor (Ross et al., 2004). Therefore, perineural outgrowth may represent another issue important for clinical practice, particularly in younger patients whose tumors are generally more aggressive, although this has not been fully confirmed in the literature yet.

The interactions between tumor and neural cells are not limited to cell migration and tumor growth from the primary location. Such communication can also stimulate axonogenesis or extent of nerves together with an increase of axon numbers, which can lead to an increase in tissue density around the neural tissue. This process is important in embryonic development and wound healing, representing a physiological status (Aller et al., 2010). Unfortunately, in oncology, axonogenesis facilitates tumor progression, as demonstrated in adenoid cystic carcinoma, which is accompanied by the release of neural growth factors into surrounding tissues. These factors, in turn, stimulated the nervous tissue to neurogenesis, causing nerve elongation, which allowed the tumor to spread further into the tissue (Wang et al., 2009). A similar phenomenon has been demonstrated in the prostate tumor, where the nerve-tumor complex has created a microenvironment stimulating both the nerve and the tumor to grow together (Ayala et al., 2008). Whether the squamous cell carcinoma also similarly affects neurogenesis remains unclear (Binmadi and Basile, 2011).

Perineural invasion is involved in the progression of head and neck squamous cell carcinomas and significantly affects disease prognosis; that is why it is critical to clarify the underlying molecular mechanisms of PNI. However, only a minimal number of molecular studies have been focused on the roles of individual genes in the regulation of the PNI process (Binmadi et al., 2012; Yu et al., 2014; Scanlon et al., 2015; Zhang et al., 2019). To our best knowledge, no one has focused on the role of epigenetic mechanisms potentially involved in regulating genes in cells participating in perineural invasion of HNSCC.

## 3 Epigenetic Regulations and Their Association with Perineural Invasion

Epigenetics is the research field focused on heritable, reversible changes caused by modulation of gene regulation and expression that do not involve changes to the underlying DNA sequence itself. The word “epigenetic” literally means “above” or “on top of” genetics (Waddington, 1942). Epigenetic processes can lead to altered gene initiation and expression, affecting cell physiology, leading to cancer transformation and progression. Epigenetic changes are dynamic and more frequent than gene mutations and potentially play an important role in determining metastatic phenotypes of cancer cells (Guo et al., 2019). It is widely accepted that transcription and gene regulation is intimately coupled to covalent modifications of the underlying chromatin template. Recently, non-enzymatic covalent modifications (NECMs) by chemically reactive metabolites have been reported to manipulate chromatin architecture and gene transcription (Zheng et al., 2019a; Zheng et al., 2020a). In turn, miRNAs as short non-coding RNAs that regulate gene expression post-transcriptionally, generally bind to the 3′-terminal untranslated region (3′-UTR) of their target mRNAs and repress protein production by destabilizing the mRNA and translational silencing (Ramzan et al., 2021).

In this review, we focus on epigenetic modifications, including classical epigenetic changes affecting DNA methylation and histone modifications and small RNA-mediated processes, particularly those of miRNAs. The link between such epigenetic changes and metastatic dissemination of OSCC by perineural invasion has not been fully explored yet. However, the specific epigenetic alterations have been previously identified in a variety of other malignancies displaying perineural invasion (PNI), such as cutaneous squamous cell carcinoma (Hervás-Marín et al., 2019), pancreatic cancer (Zhang et al., 2020), colorectal cancer (Li et al., 2019), and bile duct cancer (Park et al., 2013).

Epigenetics in oral carcinogenesis can be frequently modulated by endogenous, as well as environmental factors. Among them, tobacco smoking, alcohol consumption, and human papillomavirus (HPV) persistent infection play a significant role (Lleras et al., 2013; Kumar et al., 2015; Degli Esposti et al., 2017; Russo et al., 2020). These factors have already been linked to perineural invasion occurrence in head and neck squamous cell carcinoma (Baumeister et al., 2018; Al Feghali et al., 2019).

Epigenetic changes, such as DNA hypermethylation of tumor-suppressor genes, are abundant for OSCC while there is a limited number of oncogenic cancer-driving genetic abnormalities in this disease. Moreover, PNI represents the major clinicopathological factor affecting the poor prognosis of oral cancers. These facts encourage us to summarize current knowledge on epigenetic abnormalities during PNI.

## 4 Epigenetic Modifications of the Genes Involved in the Perineural Invasion Process in Head and Neck Squamous Cell Carcinoma from the Omics Perspective

There are multiple cellular and molecular alterations known to occur along with the perineural invasion of tumorous tissues. These changes result in the molecular profiles associated with epithelial-mesenchymal transition, metastasis, and invasion as revealed by single-cell transcriptomics in HNSCC (Zhang et al., 2019). The global expression adjustments can be accompanied by altered activity of epigenetic factors that simultaneously control the expression of many gene sets. Targeted analysis on TGCA dataset using weighted gene co-expression network approach to identify a pool of most important genes highly associated with the presence of perineural invasion revealed the following 12 candidate genes: TIMP2, MIR198, LAMA4, FAM198B, MIR4649, COL5A1, COL1A2, OLFML2B, MMP2, FBN1, ADAM12, and PDGFRB (Zhang et al., 2019). Most of these genes were highly expressed in fibroblasts, with relatively high expression observed in macrophages and endothelial cells (Zhao et al., 2014; Zhang et al., 2019). The epigenetic regulation of those genes in the OSCC patients with perineural invasion is mainly unknown. However, the epigenetic status of many of those core genes was already described in other malignancies (Table 1).

**TABLE 1:** Expression and epigenetic regulation of PNI-HNSCC signature genes.

Gene name	Expression in HNSCC/OSCC (Ref.)	Epigenetic regulation in HNSCC/OSCC (Ref.)	Epigenetic regulation in other types of cancers (Ref.)
TIMP2	controversial Shrestha et al. (2017); **↑** in HNSCC/OSCC; Ondruschka et al. (2002), Ruokolainen et al. (2006), Yoshizaki et al. (2001); **↓** in OSCC; Gao et al. (2020), Chen et al. (2019)	HPV(-) HNSCC tumors showed **hyper-5hmC**; Liu et al. (2020)	**methylation** in prostate tumor; Pulukuri et al. (2007); **methylation** in glioblastoma and breast carcinoma; Chernov et al. (2009); **EZH2** histone methylation in ovarian cancer; Yi et al. (2017)
LAMA4	**↑** in OSCC; Franz et al. (2010)		**DNA hypomethylation** in pancreatic cancer; Zheng et al. (2020b)
FAM198B	**↑** in HNSCC - fibroblasts; Zhang et al. (2019)		regulation through **RNA-binding proteins** in ovarian cancer; Guo et al. (2021)
COL5A1	↑ in OSCC Li et al. (2017)		**EZH2** histone methylation in breast cancer; Kumari et al. (2019)
COL1A2	↑ in HPSCC; Lin et al. (2020a)	**hypermethylation** in HNSCC; Misawa et al. (2011); Misawa et al. (2016)	regulated by **miR-25-3p** in pancreatic cancer; Wu et al. (2019)
OLFML2B	↑ in HNSCC- fibroblasts; Zhang et al. (2019)		**N6-methyladenosine (m6A)-modification** in rectum adenocarcinoma; Huang et al. (2021); **active enhancer-associated genes** in cirrhosis and hepatocellular carcinoma; Yang et al. (2020)
MMP2	↑ Yoshizaki et al. (2001), Ondruschka et al. (2002), Yorioka et al. (2002), Ruokolainen et al. (2006), Celentano et al. (2021)	**chromatin remodeling** in OSCC Yamamoto et al. (2020); HPV(-) HNSCC tumors exhibit **hyper-5hmC** Liu et al. (2020)	DNA **hypomethylation** in glioblastoma; Chernov et al. (2009)
FBN1	↑ in HNSCC Puram et al. (2017), Feng et al. (2021)		**hypermethylated** in colorectal cancer; Guo et al. (2013), Coppede et al. (2014)
ADAM12	↑ in OSCC Uehara et al. (2012)	regulated by **miR-29a-3p** in OSCC Jiang et al. (2021)	DNA **hypomethylation** in breast cancer; Mendaza et al. (2020)
PDGFRB	↑ in OSCC Kartha et al. (2016), Lin et al. (2020b)		**methylation** in osteosarcoma; Gentilini et al. (2020)

The expression of metalloproteinase tissue inhibitor 2 (TIMP2) was found to play a crucial role in the progression of malignant tumors, was downregulated in many cancers, including OSCC (Chen et al., 2019; Zhang et al., 2019). OSCC cell lines and tissue exhibited reduced expression of TIMP2, which was negatively correlated with CSN6 (COP9 Signalling Complex) (Gao et al., 2020). However, many studies identified high expression of TIMP2 to correlate with poor prognosis (Ruokolainen et al., 2006). This controversy of the results was already previously discussed (Shrestha et al., 2017). Nevertheless, silencing of TIMP2 by the activity of histone methyltransferase EZH2 was found to promote cell invasion and migration in metastatic ovarian cancer (Yi et al., 2017). Interestingly, another hub gene, COL5A1, as a target of EZH2, is upregulated by decreased activity of EZH2 in the breast carcinoma cell line (Kumari et al., 2019). As the expression of EZH2 was found to be increased in HNSCC, and the silencing of EZH2 led to the suppression of tumour invasion (Cao et al., 2012), this protein seems to be a promising target for therapies; however its association with PNI has to be evaluated yet. Moreover, COL1A2 is highly expressed in pancreatic cancer and represents a hallmark protein regulated by miR-25-3p (Wu et al., 2019). Hypomethylation of LAMA4 was identified as a marker of poor prognosis in pancreatic cancer (Zheng et al., 2020b). The relationship between LAMA4 expression and various clinicopathological features, including PNI, was analysed. Nevertheless, PNI was not significantly correlated with LAMA4 upregulation (*p* = 0.210). Epigenetic regulation of FAM198B is probably only indirect through its stabilization protein CELF2 (Guo et al., 2021). CELF2 is involved in mRNA splicing, and epigenetic loss of CELF2 was associated with a worse prognosis in breast cancer (Piqué et al., 2019). CELF2 was found to be differentially expressed in HNSCC, and its low expression was determined in normal head and neck tissues (Yang et al., 2021). Therefore, CELF2, similar to EZH2, was defined as a negative prognostic marker for HNSCC (Yang et al., 2021); however, the association of this cancer to PNI is still unknown and will need to be validated. For metalloproteinase 2 (MMP2), an epigenetic control by promoter hypomethylation was suggested to be associated with invasive cancer behaviour (Chernov et al., 2009). Similarly, hypomethylation of ADAM12 promoter was linked with worse outcomes in breast cancer (Mendaza et al., 2020).

Interestingly, the expression of the majority (75%) of core genes seemed to be the highest in fibroblasts (Zhang et al., 2019), in agreement with the known fact that cancer-associated fibroblasts are essential for disease progression and their potential role in directing and promoting perineural invasion will also be necessary to evaluate.

## 5 Specific Epigenetic Modifications in Head and Neck Squamous Cell Carcinoma and Other Neurotropic Cancers Associated with Perineural Invasion

Both DNA and histone modifications play a crucial role in regulating DNA transcriptional activity and chromatin structure. Those DNA and histone modifications are facilitated by epigenetic regulators (DNA methyltransferases, ten-eleven translocation 5-mC hydroxylases, histone acetyltransferases, histone methyltransferases, chromatin-remodeling enzymes, and many more), representing the executors of chromatin remodeling outcomes.

Potential links between DNA and histone modifications, or the expression of epigenetic regulators as well as short non-coding RNA, and the PNI extent have not been addressed in the HNSCC field so far. However, previously uncovered correlation of the altered expression level of epigenetic regulators and perineural invasion found in pancreatic ductal adenocarcinoma (PDA) provided the venue for developing potential therapeutic targeting these enzymes (Silverman and Shi, 2016). Those principles can also be adapted to target epigenetic regulators in PNI positive HNSCC patients.

Here, we focus on individual epigenetic changes in HNSCC and other neurotropic cancers and their possible association with PNI.

### 5.1 DNA and Histone Epigenetic Modifications Associated with Perineural Invasion

#### 5.1.1 DNA Methylation

In mammals, DNA methylation refers to the covalent transfer of a methyl (-CH3) group to the C5 position of the cytosine ring of DNA by a family of DNA methyltransferases (DNMTs). DNMT family comprises a conserved set of DNA-modifying enzymes DNMT1, DNMT3A, and DNMT3B (Li and Zhang, 2014). Most of DNA methylations in somatic cells occur in the CpG dinucleotide context. The cluster of CpG dinucleotides, referred to as CpG islands, is commonly found near transcription start sites (TSS) of many genes. DNA methylation negatively correlates with gene expression, leading to the transcriptional silencing of genes controlling cancer progression.

As mentioned above, HNSCC is characterized by dysregulation of tumor-suppressor genes, primarily by epigenetic abnormalities (Faraji et al., 2018). CpG islands near TSSs of tumor suppressors are often hypermethylated in the oral squamous cell carcinoma (reviewed in Kim et al., 2019; Hema et al., 2017). DNA methylation status of these OSCC-specific tumor-suppressor genes was not studied specifically concerning PNI in oral cancers. Nevertheless, the differential expression of some of them, such as TFPI2, CDKN2A, CDH1, PTEN, and RUNX3, correlated with PNI in other neurotropic cancers (Supplementary Table S1).

TFPI2 (Tissue factor pathway inhibitor-2), Kunitz-type serine proteinase, and a presumed tumor suppressor gene was associated with PNI in pancreatic carcinoma tissue. TFPI2 expression was strongly negatively correlated (r = −0.460, *p* < 0.001) with PNI and proposed to be a predictor of a high risk of PNI in pancreatic cancer (Zhai et al., 2015). Moreover, aberrant methylation of the TFPI2 gene is significant in the decrease of TFPI2 expression in human cancers in general (Sierko et al., 2007). However, the association of the methylation status of these genes with PNI in OSCC has not been tested yet.

CDKN2A (cyclin-dependent kinase inhibitor 2A) is ubiquitously expressed in many tissues and cell types and codes for two tumor suppressor proteins. These are transcribed from alternative first exons and therefore translated from different reading frames, one of them being p16INK4a (p16), the other one p14ARF (p14) (Stott et al., 1998). In OSCC, CDKN2A loss of expression was associated with the disease recurrence (Deepak Roshan et al., 2019), and p16 as one of the well-accepted markers of HNSCC prognosis positively correlated with better disease outcomes (reviewed in Augustin et al., 2020). Association of OSCC recurrence and PNI was also tested but determined to be insignificant (Deepak Roshan et al., 2019). Remarkably, methylation of p16INK4a promoter was a more frequent event in prostatic tumors with PNI than without PNI (Verdoodt et al., 2011).

The adhesion molecule and tumor suppressor gene CDH1 (E-cadherin) was found to be frequently inactivated in salivary gland ACC and ACC cell lines through promoter methylation. Tumors with CDH1 promoter methylation exhibited a significantly more PNI than tumors, not demonstrating methylations (Zhang et al., 2007).

Loss of PTEN (phosphatase and tensin homolog), a commonly altered tumor suppressor gene in prostate cancer, was analyzed in prostatic adenocarcinoma specimens from patients who subsequently developed biochemical recurrence. PTEN loss demonstrated a significant correlation with perineural invasion (RR = 24.489, *p* < 0.001), as compared to wild-type PTEN (Kim et al., 2015). Interestingly, prostate tumors with PTEN loss harbored a distinct epigenome-wide methylation signature, which might mediate tumor progression when PTEN was deleted (Geybels et al., 2017).

The tumor regulatory role of RUNX3 (runt-related transcription factor 3), the Wnt pathway antagonist, in OSCC is still controversial. It was previously identified as a tumor suppressor regulating OSCC cells invasion (Zhou et al., 2017). Moreover, its promoter was found hypermethylated in OSCC (Gao et al., 2009; Supic et al., 2011). However, it may function either as an oncogene or a tumor suppressor gene, based on the data for other tumor types (Otálora-Otálora et al., 2019). Unfortunately, the reduction of this transcription factor has not been studied in relation to PNI in OSCC yet. Nevertheless, the correlation of RUNX3 methylation and PNI was reported in salivary gland adenoid cystic carcinoma (ACC) (Ge et al., 2011), indicating the necessity to analyze RUNX3 methylation in other types of neurotropic cancers.

In addition to tumor suppressor genes, methylation of the growth factor receptors such as TrkA, GFRA1, and TNFRSF10C were identified to play a role in perineural spreading and invasion in some tumors (Supplementary Table S1).

Neurotrophic tyrosine kinase receptor type 1 (NTRK1), also known as tropomyosin receptor kinase A (TrkA), is a high-affinity catalytic receptor for the nerve growth factor (NGF). NGF-TrkA signaling system is thought to be involved in the progression of various cancers (reviewed in Gao et al., 2018) and was suggested to play a role in perineural growth and invasion in HNSCC (Roh et al., 2015). An enhanced expression of the NGF and TrkA in pancreatic cancer was considered to be related to the perineural invasion of cancer cells (Zhu et al., 1999). Immunohistochemical analysis revealed strong TrkA expression in most stage IV pancreatic ductal cancer cells, especially with extensive perineural invasion. Intriguingly, TrkA expression was positively related to the methylation of non-CpG islands around the negatively regulating AP-1-like site in the 5′-untranslated region of the TrkA gene (Fujimoto et al., 2005).

GFRA1 (GDNF family receptor alpha-1) is a cell surface membrane receptor for glial cell-derived neurotrophic factor (GDNF). The GFRA1 gene is normally expressed in neural cells and is overexpressed in many cancer types. Methylation of CpG islands around the GFRA1 transcription start site epigenetically inactivates gene transcription, and demethylation of the locus is essential for gene reactivation. Interestingly, GFRA1 overexpression promotes perineural invasion in pancreatic (Gil et al., 2010) and bile duct cancer cells (Iwahashi et al., 2002).

Higher methylation levels at TNFRSF10C (tumor necrosis factor receptor superfamily member 10C) promoter region in peripheral blood and cancer tissue was positively associated with perineural tumor spread in pancreatic adenocarcinoma (Dauksa et al., 2012); however, it will be necessary to follow the gene expression regulation of this gene in HNSCC patients.

As of today, the only direct correlation of gene methylation with perineural invasion in HNSCC was reported for homeobox protein HOXA1 (Li et al., 2021). High HOXA1 expression was significantly related to DNA methylation decline and PNI (*p* = 0.0019) (Li et al., 2021). Therefore, HOXA1 was proposed to be a novel biomarker of HNSCC prognosis.

#### 5.1.2 Methylation and Demethylation Enzymes

Modulation of the DNA methylation status may be reflected by the expression changes of the methylation and demethylation enzymes. Such changes in the level and activity of DNMTs and TET (ten-eleven translocation 5-mC hydroxylases) enzymes may contribute to OSCC initiation, progression, and clinical outcome. The gene expression data on DNMTs and TETs concerning PNI have been reported (Table 2).

**TABLE 2:** Expression of methylation and demethylation enzymes and their association with PNI.

Gene name	Expression in HNSCC/OSCC	References
DNMT1	↑ in OSCC	Supic et al. (2017); Gaździcka et al. (2020)
DNMT3A	↑ in OSCC	Gaździcka et al. (2020)
DNMT3B	↑ in OSCC	Gaździcka et al. (2020)
TET-1	↓ in HNSCC	Thienpont et al. (2016); Misawa et al. (2018)
TET-2	↓ in HNSCC	Jäwert et al. (2013); Thienpont et al. (2016); Wang et al. (2017)
TET-3	↓ in HNSCC	Thienpont et al. (2016); Misawa et al. (2018)
HDAC2	↑ in OSCC	Chang et al. (2009)
HDAC6	↑ in OSCC	Sakuma et al. (2006)
HDAC8	↑ in OSCC	Ahn et al. (2017)
HDAC9	↑ in OSCC	Rastogi et al. (2016)
CAF1/p60	↑ in OSCC	Staibano et al. (2007); Mascolo et al. (2012)
EZH2	↑ in HNSCC	Cao et al. (2012)

The mRNA expression of all DNMT family members was upregulated in OSCC (Gaździcka et al., 2020). Thus, upregulation of the DNMT1 gene was confirmed to be an independent marker of relapse-free survival and poor clinical outcome of OSCC patients (Supic et al., 2017).

Overexpression of DNMT3A in OSCC was related to the downregulation of the anti-aging gene Klotho and may be one of the causes of carcinoma in the oral and maxillofacial region. Klotho may serve as a reliable marker for early detection of methylation changes in oral tissues or can be used as a potential target for therapeutic modification in OSCC (Adhikari et al., 2017).

Increased expression of DNMT3B leads to downregulation of E-cadherin, suggesting that changes in OSCC methylation status are involved in the induction of epithelial-mesenchymal transition (EMT) (Chen et al., 2016). Furthermore, in gene expression profile study of salivary ACC associated with PNI, DNMT3B was listed among substantially upregulated genes in the PNI ACC cell group relative to the non-PNI ACC cell group (Chen et al., 2007). Together, these findings agree with the detected E-cadherin promoter methylation in PNI-positive salivary gland ACC discussed in the previous chapter (Chapter 5.1.1 and Zhang et al., 2007).

Importantly, it was shown that targeting DNMTs using epigenetic inhibitors might potentially reverse methylated status and therefore enhance OSCC response to chemotherapy (Suzuki et al., 2009).

The process of active removal of methyl groups is opposite to DNA methylation. The TET enzymes, TET-1, TET-2, and TET-3, play a role as pivotal factors in DNA demethylation. The TET enzymes catalyze the hydroxylation of DNA 5-methylcytosine (5mC) to 5-hydroxymethylcytosine (5hmC) and can alter the regulation of transcription (Rasmussen and Helin, 2016) when molecular oxygen is served as a substrate to convert 5-mC to 5-hmC, and 5-hmC to 5-formylcytosine (5-fC) and 5-carboxycytosine (5-caC) (Ito et al., 2011; Ko et al., 2015). Furthermore, the discovery of 5-hmC, 5-fC, and 5-caC has raised the need to elucidate their function, and it is possible that such oxidized cytosine modifications constitute part of the pathways that lead to active demethylation (Booth et al., 2015).

TET proteins are involved in many important processes, which may influence the development and progression of tumorigenesis (Tan and Shi, 2012). In OSCC, high levels of 5-hmC were correlated with poor overall survival in patients, suggesting the level of demethylation as an important factor for disease prognosis (Wang et al., 2017) and were associated with perineural invasion in pancreatic ductal adenocarcinoma (Chen et al., 2018).

A downregulation of TET-1 expression may lead to enhanced O6-methylguanine-DNA methyltransferase (MGMT) promoter methylation, increasing the sensitivity of OSCC stem cells to chemotherapeutics (Wang et al., 2019). Furthermore, a downregulation of TET-2 correlated with a decreased level of 5-hmC in patients with OSCC (Jäwert et al., 2013). Thus, the reduced abundance of 5-hmC and the depletion of TET-2 expression in OSCC patients may contribute to OSCC development (Wang et al., 2017).

In addition, the multivariate analysis revealed the association of TET-3 gene methylation with poor survival of OSCC patients (Misawa et al., 2018); however, the direct effect of this epigenetic change on perineural invasion initiation in HNSCC patients has not been evaluated yet (Table 2).

#### 5.1.3 Histone Modifications

Histone post-translational modifications, not limited to acetylation, methylation, and phosphorylation, represent distinct types of epigenetic regulation, which can be altered during cancer progression. A high level of H3K27me3 and a low level of H3K4ac were positively correlated with PNI and OSCC tumor stage (Chen et al., 2013). In OSCC tissue and cell lines, H3K27 acetylation promoted cell proliferation and invasion via the activation of the Wnt/βcatenin pathway (Chen et al., 2019). Furthermore, H3K14 was found to be highly acetylated in OSCC tumor patient samples compared to adjacent normal tissue (Arif et al., 2010).

Overexpression of chromatin assembly factor-1, CAF1/p60, which is implicated in incorporating H3K56-acetylated histones into chromatin in response to oxidative stress, predicts the metastasizing behavior of oral cancer (Mascolo et al., 2012).

A low level of H3K4me2 was associated with perineural invasion (*p* = 0.07) in Asian patients with pancreatic cancer (Watanabe et al., 2012). Potentially, a low level of H3K4me2 may influence PNI in HNSCC patients, but this possibility was not evaluated yet.

#### 5.1.4 Histone Deacetylation Enzymes

Histone deacetylases (HDACs) are involved in various cellular functions, including cell survival and proliferation regulation. Therefore, aberrant expression of HDAC genes is often implicated in tumorigenesis (Hadley et al., 2019); specifically the overexpression of HDACs is usually associated with advanced OSCC (Chang et al., 2009).

The elevation of HDAC2 expression was frequently found in OSCC patients, and univariate analysis confirmed the association of HDAC2 overexpression with shorter overall survival of those patients. This data suggests that the expression level of HDAC2 can serve as a useful prognostic marker for patients with OSCC (Chang et al., 2009).

Higher mRNA and protein expression of HDAC6 were detected in oral cancers, and these changes were associated with a level of tumor aggressiveness (Sakuma et al., 2006).

HDAC8 was found to be overexpressed in OSCC tissues, and HDAC8 silencing significantly inhibited the proliferation of OSCC cells by the induction of apoptosis through caspases activation and pro-survival autophagy (Ahn and Yoon, 2017). In breast cancer tissue, there was a significant association between HDAC8 overexpression and perineural invasion (*p* < 0.05) (Menbari et al., 2020).

High expression of HDAC9 enhances the development of OSCC by alterations of the cell proliferation, cell cycle, and apoptosis (Rastogi et al., 2016).

Based on these data, chemical inhibition of histone deacetylase members might become a novel therapeutic strategy for OSCC. Indeed, the treatment of OSCC with histone deacetylase inhibitors revealed promising results (Sakuma et al., 2006; Tasoulas et al., 2015; Ahn and Yoon, 2017).

### 5.2 Short Non-Coding RNAs Associated with Perineural Invasion

MicroRNAs (miRNAs or miRs) are a widespread class of short non-coding RNAs, approximately 18–25 nucleotides in length, which are now recognized as one of the major regulators of gene families expression in eukaryotes (Yao et al., 2019). The roles of miRNAs in epigenetic regulation are complex. However, their primary function lies in the binding to complementary target sequences in mRNA, inducing mRNA decay and interfering with the translational process, thereby preventing or modifying the translation of the protein product. As epigenetic modulators, miRNAs affect the protein levels of the target mRNAs without altering the gene sequences. Moreover, miRNAs themselves can also be epigenetically regulated by DNA methylation and histone modifications of their promoters or by epigenetic modification of the miRNA itself. The reciprocal actions of miRNAs and epigenetic pathways appear to form a miRNA-epigenetic feedback loop (Yao et al., 2019). In miRNA-mediated regulatory networks, one miRNA can regulate many genes, and a single gene can be controlled by many miRNAs (Jonas and Izaurralde, 2015).

**FIGURE 2:**
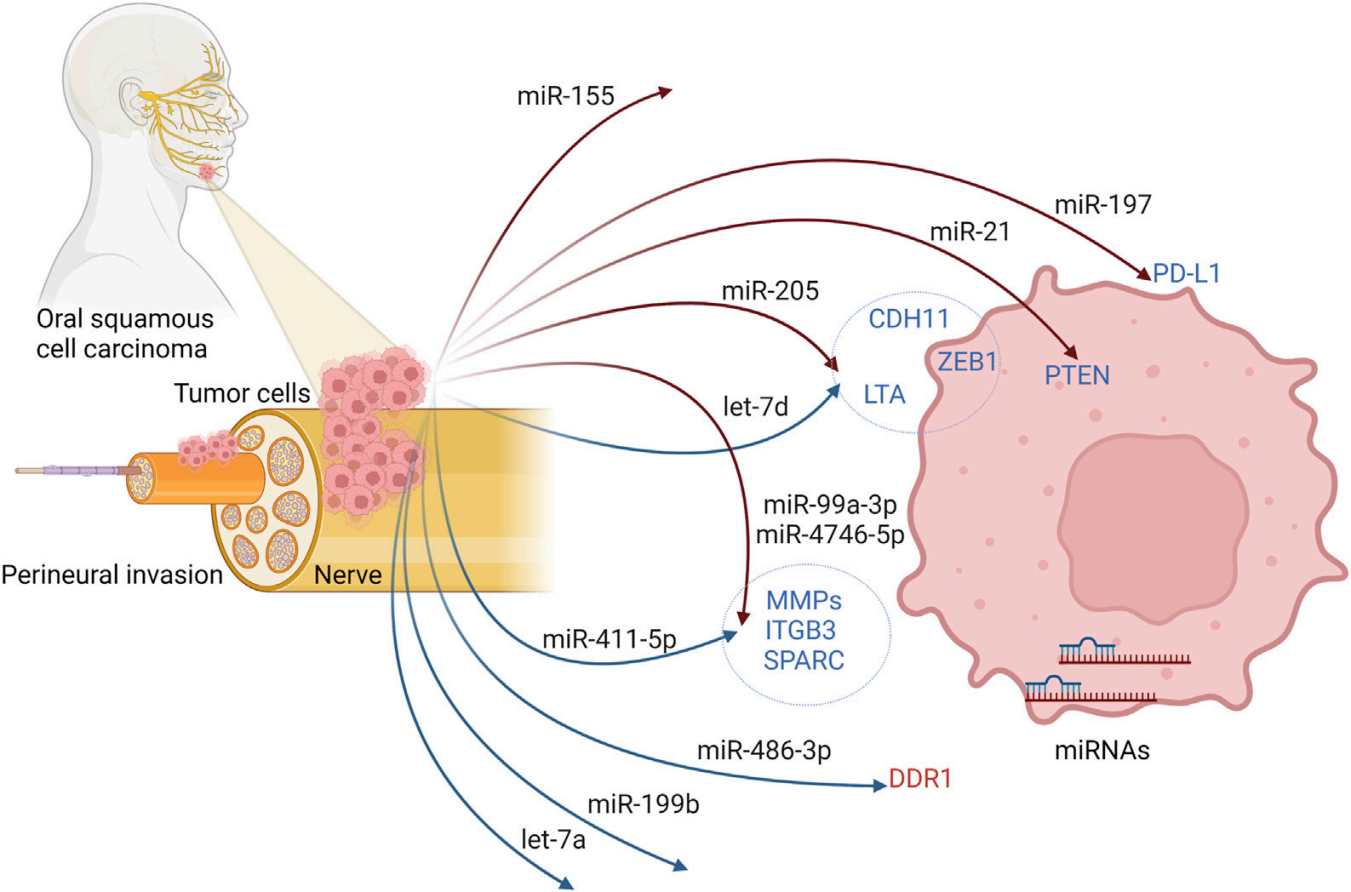
Possible regulatory mechanism of miRNAs and their targeted genes to initiate PNI in oral squamous cell carcinoma. Created with BioRender.com.

miRNA expression patterns have been studied in HNSCC patients and demonstrated to play an essential role in HNSCC pathogenesis. However, not much attention has been paid to the association of specific miRNA expression with PNI in HNSCC (Table 3; Figure 2), in contrast to other tumor types (Zhang et al., 2021). There are more studies focused on the differences in miRNAs expression between PNI positive and negative patients with prostate, pancreatic, colorectal, or gallbladder carcinoma (Prueitt et al., 2008; Liu et al., 2018; Fukada et al., 2020; Sim et al., 2020; Szabo et al., 2020). To the best of our knowledge, a small number of miRNAs have been studied in HNSCC in relation to PNI (Table 3).

**TABLE 3:** Short non-coding RNAs associated with PNI.

miRNA	Expression in HNSCC	Affected molecule (expression)	Association with PNI	References
miR-21	OSCC (↑)	PTEN (↓)	significant association with PNI	Yu et al. (2017)
	OSCC (↑)	-	the independent prognostic factor for disease-free survival together with PNI (borderline significance) in multivariate analysis	Hedbäck et al. (2014)
	OSCC (↑)	-	significant association with tumor local invasion	Mahmood et al. (2019)
miR-197	OSCC (↑)	PD-L1 (↓)	PNI is not significantly different between miR-197low and miR-197high subgroup or between low PD-L1 and high PD-L1 subgroups	Ahn et al. (2017)
miR-486-3p	OSCC (↓)	DDR1 (↑)	high expression of DDR1 significantly related to perineural invasion	Chou et al. (2019)
miR-205; let-7d	HNSCC (↑); (↓)	CDH11 (↓), ZEB1 (↓), LTA (↓)	significant difference between miR-205 level with regard to PNI	Kolenda et al. (2019)
miR-99a-3p; miR-411-5p;; miR-4746-5p	HPV16 + HNSCC (↑)	MMP1 (↓), MMP2 (↓), MMP3 (↓), MMP7 (↓), MMP9 (↓), MMP11 (↓), MMP12 (↓), MMP13 (↓), MMP16 (↓), MMP28 (↓)	significantly low frequent PNI	Zhang et al. (2020)
	(↓)	ITGB3 (↓), SPARC (↓)		
	(↑)			
let-7a	Oral cavity and oropharynx SCC (↓)	-	significant association with PNI	Brito et al. (2016)
miR-199b	HNSCC (↓)	-	significant association with PNI	Sousa et al. (2016)
miR-155	OSCC (↑)	-	association with PNI (borderline significance)	Rajan et al. (2021)

An extensive effort has been devoted to revealing direct (Yu et al., 2017) and indirect (Reis et al., 2010; Hedbäck et al., 2014; Mahmood et al., 2019) correlation of tumor progression with the miR-21 expression status in OSCC. miR-21 is one of the most studied oncogenic microRNAs in the field of carcinogenesis (Feng and Tsao, 2016), and it represents the most consistently dysregulated microRNA in OSCC (reviewed in Narasimhan and Narasimhan, 2018). Interestingly, high expression of miR-21, reversely correlated with PTEN expression, was related to perineural invasion and was suggested to promote cancer cells to invade the nerve bundle and spread out (Yu et al., 2017).

The effects of altered miR-197 expression on various clinicopathological features in the prognosis of OSCC were identified (Ahn et al., 2017). Increased level of miR-197 was found to be associated with gender, T stage, and PD-L1 (Programmed Death Ligand 1) expression changes. However, the association of miR-197 expression with PNI was not statistically significant (Ahn et al., 2017). In addition, the inverse correlation of miR-486-3p and DDR1 (Discoidin Domain Receptor Tyrosine Kinase 1) expression was reported in OSCC tissues, and clinical analysis uncovered the association of high expression of DDR1 with perineural invasion (Chou et al., 2019).

Increased expression of miR-205 was linked to less aggressive HNSCC tumors with a lower ability of perineural invasion (Kolenda et al., 2019). In that study, the expression of two tumor suppressors and regulators of epithelial-to-mesenchymal transition, let-7d and miR-205, were investigated together. Only high expression of miR-205 significantly inversely correlated with perineural invasion. Moreover, LTA, ZEB1, and CDH11 were found to be down-regulated in the group of patients with low let-7d and high miR-205 compared to patients with high let-7d and low miR-205 expression levels.

In another study with a relatively small group of HNSCC patients, a couple of miRNAs were analyzed in association with lymph node and perineural invasion (Sousa et al., 2016). In that study, the researchers found a significant association of reduced miR-199b levels with PNI (*p* = 0.040).

Specifically, in HPV16 + HNSCC subtype, miR-99a-3p high, miR-411-5p low, miR-4746-5p high expression phenotype correlated with increased overall survival and less frequent PNI (Zhang et al., 2020). This was probably caused by deregulation of EMT-related signaling and invasion-related genes in miR-99a-3p high, miR-411-5p low, miR-4746-5p high HNSCC patients.

In a broader category of HNSCC, downregulation of let-7a, miRNA involved in stem cell regulation, was significantly associated (*p* = 0.042) with perineural invasion (Brito et al., 2016).

Recently, a well-known oncogenic miR-155 was reported to be potentially valuable as a novel therapeutic target (Rajan et al., 2021). miR-155 was found to be overexpressed in OSCC patients exhibiting metastases into neck lymph nodes, and correlation of miR-155 expression with perineural invasion was statistically significant (Rajan et al., 2021). Earlier, a product of pre-miR-155 from the 5′ arm, miR-155-5p, was suggested to be a factor of poor disease-free survival rate and poor prognosis in OSCC (Baba et al., 2016). Moreover, in OSCC patients, enhanced overexpression of miR-155 inhibited the expression of the CDC73 gene, promoted cell proliferation, and inhibited apoptosis (Rather et al., 2013).

**FIGURE 3:**
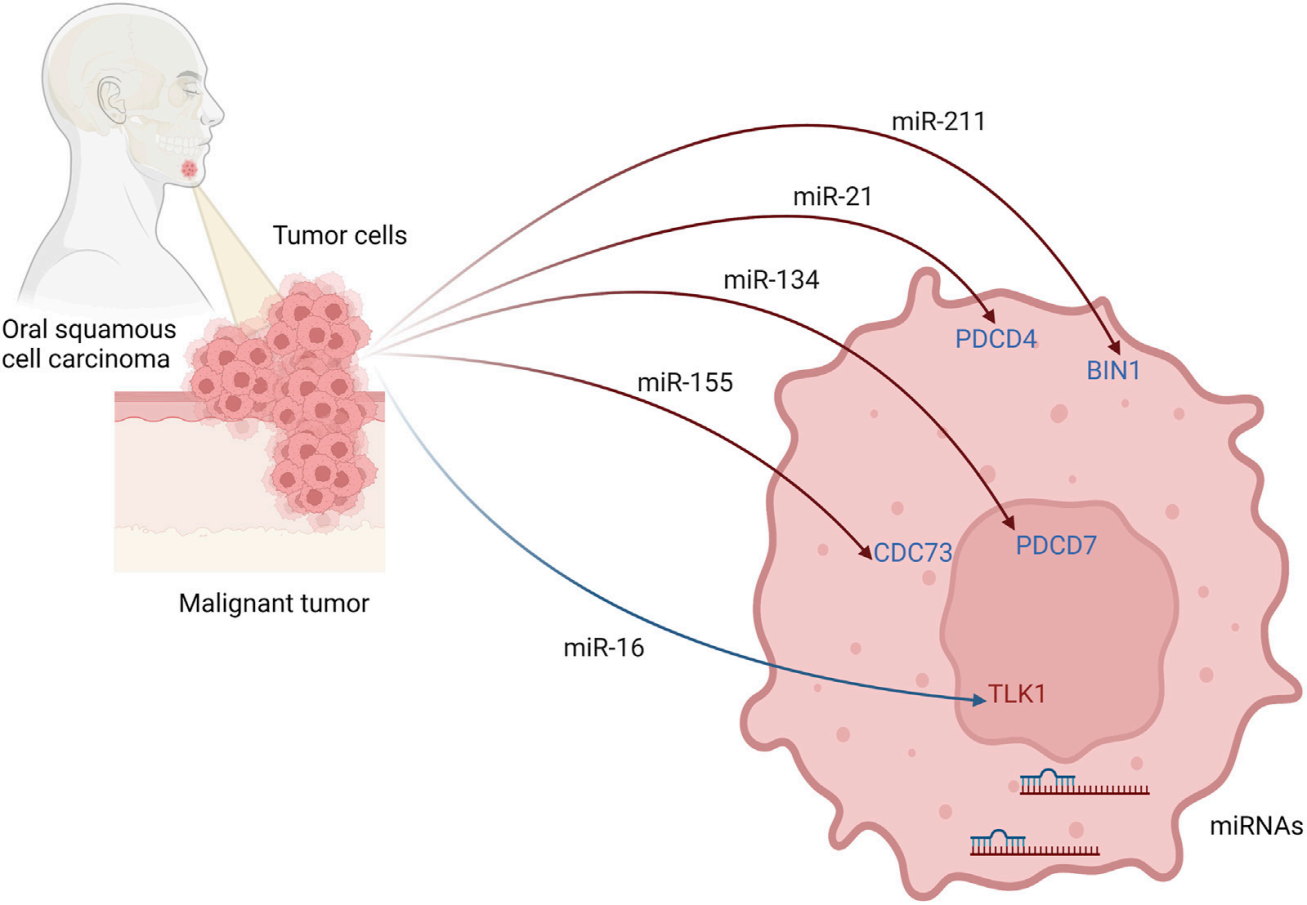
Possible regulatory mechanism of miRNAs and their targeted genes to initiate invasive behaviour of oral squamous cell carcinoma. Created with BioRender.com.

Several other miRNAs have been studied in oral squamous cell carcinoma cell lines and correlated with more aggressive characteristics and metastasizing behavior of OSCC in general (Table 4; Figure 3). The level of miR-21 was increased in OSCC patients with nodal metastasis (Reis et al., 2010). Its higher expression correlated with the downregulation of Programmed Cell Death 4 (PDCD4), the tumor suppressor genes, and the increased invasive potential of oral carcinoma cells.

**TABLE 4:** Short non-coding RNAs associated with malignant behavior in OSCC.

miRNA	Expression in OSCC	Affected molecule (expression)	Associated malignant behavior of human OSCC cells	References
miR-21	(↑)	PDCD4 (↓)	PDCD4 over-expression in primary OSCC cells decreased percentage of invading cells; knock-down of PDCD4 increased the number of invading cells	Reis et al. (2010)
miR-211	(↑)	BIN1 (↓)	miR-211 inhibition and BIN1 overexpression suppressed OSCC cell proliferation, migration, and invasion ability, and enhanced apoptosis	Zheng et al. (2019b)
miR-134	(↑)	PDCD7 (↓)	exogenous PDCD7 expression reduced growth and migration of OSCC cell line; PDCD7 knockout increased migration and decreased CDH-1 mRNA in OSCC cell lines	Peng et al. (2018)
miR-155	(↑)	CDC73 (↓)	decreased proliferation and enhanced apoptosis in miR-155 antagonist treated OSCC cell line overexpressing miR-155; significant reduction in tumor volume and the weight formed by miR-155 antagonist pretreated OSCC cell line overexpressing miR-155	Rather et al. (2013)
miR-16	(↓)	TLK1 (↑)	forced expression of miR-16 in OSCC cell lines inhibited cell proliferation *in vitro* and tumor growth *in vivo* by inhibition of TLK1	Hu et al. (2018)

The increased expression of oncogenic miR-211 in OSCC correlated with decreased expression of another tumor suppressor gene, bridging integrator-1 (BIN1) (Zheng et al., 2019b). Indeed miR-211-binding was found on the 3′-untranslated region (3′-UTR) of BIN1 mRNA. Moreover, the ectopic overexpression of BIN1 protein in OSCC cell lines was associated with decreased proliferation, cell migration, and invasion (Zheng et al., 2019b).

Furthermore, upregulation of miR-134 was determined in OSCC cell lines and found to target and reduce expression of the PDCD7 gene, which led to OSCC progression (Peng et al., 2018).

Downregulation of miR-16 expression was observed in OSCC patients and cancer cell lines, and this was negatively correlated with enhanced expression of its target gene, Tousled-like kinase 1 (TLK1) (Hu et al., 2018). Moreover, oncogenes AKT3 and BCL2L2, which are responsible for promoting cell proliferation and inhibition of apoptosis in OSCC, have been determined as new target genes for miR-16 with a negative correlation between expression of miR-16 and expression of AKT3 and BCL2L2 (Wang and Li, 2018). Plus, a synergic effect of miR-15a, miR-16, and miR-132 overexpression caused suppression of proliferation, migration, and invasion in pituitary tumors (Renjie and Haiqian, 2015), Decreased expression of miR-638 in OSCC tissue and cells correlated with lymph node metastasis and TMN stages (Tang et al., 2019). Ectopic expression of miR-638 in OSCC cell lines inhibited migration, invasion, and proliferation, while its knockdown had an opposite outcome. miR-638 targets phospholipase D1 (PLD1) and thus may inhibit Wnt/β-catenin pathway (Tang et al., 2019).

Overall, the involvement of microRNAs in the PNI has gained increased attention recently (reviewed in Zhang et al., 2021). The recent data suggest that microRNAs could serve as diagnostic and prognostic markers, as well as a therapeutic target for HNSCC patients.

## 6 The Influence of Other Epigenetic Factors on the Head and Neck Squamous Cell Carcinoma Incidence and Perineural Invasion

Specific and general epigenetic factors represent risk components for head and neck squamous cell carcinoma, such as tobacco use and alcohol consumption. These addictive substances are independent risk factors but exert synergistic effects when combined. Indeed, there is a behavioral link between cigarette smoking and drinking alcohol; thus, addressing each habit as a separate risk factor in the patient is not always easy. Moreover, exposure to second-hand cigarette smoke is difficult to quantify, leading to patients’ background misclassification.

While HNSCC is traditionally considered a disease of smokers and drinkers, non-smoking and non-drinking patients also develop HNSCC. Another factor that can raise a person’s risk of head and neck cancer is HPV infection. HPV-positive HNSCC represents a distinct group of lesions that vary in their clinical presentation from those caused by classical risk factors (Aggarwal et al., 2020).

Nevertheless, all of the above-listed risk factors can influence or function through epigenetic mechanisms in head and neck squamous cell carcinoma (Colacino et al., 2013; Virani et al., 2015; Degli Esposti et al., 2017; Hema et al., 2017; Ghantous et al., 2018; Jiang et al., 2019; La Rosa et al., 2020). Therefore, it is critical to address whether these mechanisms also contribute to the occurrence of perineural invasion in HNSCC.

### 6.1 Tobacco as an Epigenetic Factor of Head and Neck Squamous Cell Carcinoma

Various epidemiological studies state that 60–95% of patients with oral squamous cell carcinoma have a personal history of using tobacco products (Llewelyn and Smoking, 1994; Jaber et al., 1999), making tobacco abuse one of the essential factors affecting etiopathogenesis. Tobacco risk factors include complex cigarette smoke, individual chemical components of cigarette smoke, process of cigarette combustion, and the use of non-cigarette tobacco products. It was demonstrated that tobacco in its chewable and smoking form contains a long list of potential carcinogens, cocarcinogens, and tumor promoters. The mechanisms of action of these compounds vary and can evoke several epigenetic changes, including enzymatic hypermethylation of promoter regions of genes ultimately leading to their silencing, altered methylation patterns in gene bodies and introns, alteration of miRNAs and long non-coding RNAs, and changes in histone modifications (reviewed in Centers for Disease Control and Prevention 2010; Ghantous et al., 2018; Choukrallah et al., 2019).

Carcinogens in cigarette smoke are regarded as one of the most potent environmental modifiers of DNA methylation (Breitling et al., 2011). They can give rise to DNA double-stranded breaks. Such damage is repaired by a coordinated action of DNA repair and checkpoint systems, including maintenance DNA methyltransferase 1 (DNMT1). It is recruited to the repair sites to methylate CpGs adjacent to the repaired nucleotides. Cigarette smoke modulates the expression and activity of DNMT1, thus influencing established DNA methylation patterns in cells. Further, nicotine binds to and activates the nicotinic acetylcholine receptors (nAchRs) (Brunzell et al., 2015). nAchRs are found abundantly in the central and peripheral nervous systems. Their activation ultimately leads to downstream activation of cAMP response element-binding protein (CREB), a key transcription factor for many genes (Lenz et al., 2010). Acting possibly via this mechanism, nicotine has been shown to downregulate DNMT1 mRNA and protein expression in neurons in the mouse brain (reviewed in Lee and Pausova, 2013).

Besides DNA methylation, cigarette smoke influences nucleosomal remodeling via histone acetylation, methylation, phosphorylation, and ubiquitination. Further, it can cause abnormal expression of unstable single-stranded miRNAs and lncRNA in mice and humans (Lee and Pausova, 2013). The role of epigenetic factors in tobacco-associated carcinogenesis is further supported by the reversibility of cancer risk after cigarette smoking cessation (Guida et al., 2015).

Only a handful of studies have evaluated the association of tobacco use and PNI in HNSCC patients, and the findings are somewhat inconsistent. Baumeister et al. concluded on the set of 178 HNSCC patients that current and former smokers demonstrated PNI significantly more often than tumors of never smokers (Baumeister et al., 2018). Al Feghali et al. uncovered on the set of 163 patients with oral cavity squamous cell carcinoma that smokers were more likely to have PNI than non-smokers (*p* < 0.01) (Al Feghali et al., 2019). On the other hand, smoking history was found to be not a risk factor for PNI in 178 patients with HNSCC examined (Zhang et al., 2019). Moreover, smoking history was not associated with perineural invasion in large cohort study of 136 HPV-positive OPSCC (Zebolsky et al., 2021).

Recently, genomic signature analyses and a DNA copy number variations (CNV) analysis of OSCC from patients who self-reported no smoking and drinking habits uncovered epidermal growth factor receptor (EGFR) oncogene amplification as the most common CNV in the examined individuals (Koo et al., 2021). Furthermore, EGFR amplification was significantly associated with PNI and extracapsular spread and non-smoking and non-drinking status as well. Increased EGFR gene copies in OSCC were also positively correlated with PNI in another study (Huang et al., 2012). Interestingly, a network of epigenetic factors that directly control EGFR DNA amplification was identified lately (Clarke et al., 2020). Collectively, these findings suggest that potentially epigenetically modified EGFR oncogene level in PNI positive OSCC might be independent of smoking and/or drinking alcohol.

### 6.2 Alcohol as an Epigenetic Factor of Oral Squamous Cell Carcinoma

The association between alcohol abuse and the development of OSCC has been previously described and discussed by many authors. The relative risk factor of daily drinking of 100 g of alcohol for SCC development is 6.0 for oral cavity, compared to 4.2 for esophageal and 3.2 for laryngeal cancer (Bagnardi et al., 2001; Morse et al., 2007; Li et al., 2014). Adverse effects of chronic exposure of the oral mucosa to alcohol lead to epithelial atrophy, reduction of the basal layer cell size, and stimulation of stem cells proliferation in the oral epithelium, which, together with chronic inflammation, helps to create an environment favorable for carcinogenesis (Li et al., 2014; Liu et al., 2015).

Alcohol is also transferred by blood into the saliva and subsequently microbially oxidized to acetaldehyde. However, its subsequent transformation into acetate is limited, and acetaldehyde remains in the saliva (Seitz and Stickel, 2007). Further, alcohol-associated acetaldehyde exposure may occur in the oral cavity independently from liver metabolism. Some alcoholic beverages naturally contain acetaldehyde produced by yeasts and acetic acid bacteria and acetaldehyde from coupled auto-oxidation of ethanol and phenolic compounds (Stornetta et al., 2018). In agreement, relatively high acetaldehyde concentration was found in the saliva of OSCC patients who were smokers, alcohol abusers, and displayed poor oral hygiene. Apart from its genotoxic effect, acetaldehyde was shown to cause epigenetic histone modifications in hepatocytes (Shukla et al., 2007) and specific epigenetic modifications in neuroblastoma cell lines (D'Addario et al., 2011). However, a direct effect of the presence of acetaldehyde or ethanol on the development of the oral squamous cell carcinoma or perineural invasion has not been sufficiently proven yet (Salaspuro, 2003; Seitz et al., 2004).

Nevertheless, ethanol-associated modifications of epigenetics have been investigated in OSCC. For example, exposure to ethanol increased acetylation in the H3K9/14 and H3K27 and methylation in H3K27 and H3K9 related to an inferior survival prognosis, increased occurrence of metastases, and OSCC recurrence (Urvalek et al., 2015). In addition, in the patients with higher alcohol consumption, a higher rate of hypermethylation of the promotor region of five tumor-suppressor genes (P16, DAPK, APC, CDH1, and MGMT) was found in association with OSCC (Supic et al., 2009).

In HNSCC patients, miR-30a, miR-934, miR-3164, and miR-3178 were upregulated in oral keratinocytes exposed to ethanol and acetaldehyde. The consequence of miR-30a and miR-934 dysregulation was studied in normal and HNSCC cell lines. Induction of cell proliferation and anti-apoptotic Bcl2 gene expression were detected (Saad et al., 2015). Alcohol-associated changes in lncRNAs were also reported in OSCC. Namely, dysregulation of lnc-PSD4-1 and lnc-NETO1-1 (Yu et al., 2016).

However, a causal link between alcohol and PNI has not been studied yet. To our knowledge, the data taking into account alcohol consumption in PNI positive patients are still limited and controversial. Lee et al. tested prognostic implications for OSCC patients with two distinct forms of perineural invasion, intratumoral (IPNI) and extratumoral (EPNI). In this study, patients with EPNI, compared to patients with IPNI, had a higher prevalence of preoperative alcohol consumption (Lee et al., 2019). However, other studies demonstrated no association of alcohol history with perineural invasion in HNSCC (Zhang et al., 2019) and HPV-positive oropharynx squamous cell carcinoma (Zebolsky et al., 2021).

### 6.3 Relationship of Alteration in the Oral Microbiome as an Epigenetic Factor with OSCC and Perineural Invasion

Another local factor possibly affecting the carcinogenesis in the head and neck area is the oral microbiome, which is usually associated with pH alteration in the oral cavity. Alcohol and tobacco abuse are two of the most common causes of such microbiome changes (Chattopadhyay et al., 2019). Bacteria *Porphyromonas gingivalis* and *Fusobacterium nucleatum* produce inflammatory cytokines, induce cell proliferation, inhibit apoptosis and affect cell migration. These changes in cell behavior can further support processes leading to carcinogenesis in the oral cavity. Bacteria species (e.g., *Lactobacillus gasseri* and *Lactobacillus vaginalis*, *Fusobacterium nucleatum*) can even be used for general oral health screening, diagnosis of early changes in the oral epithelium, and prediction of chemoprevention in OSCC patients (Chattopadhyay et al., 2019).

Information on oral microbial dysbiosis in PNI-positive carcinomas is minimal. However, the association of gut microbiota composition with perineural invasion was discussed in colorectal carcinoma (Kinross et al., 2017; Scott et al., 2019), and a systematic review of metagenomic studies on the oral microbiome in oral cancer indicate the need to test the correlation of perineural invasion with oral microbiome (Su Mun et al., 2021).

### 6.4 HPV Infection as an Epigenetic Factor Associated With OSCC and Related Perineural Invasion

HPV infection has also been found to induce malignant tumors in the orofacial region (Hübbers and Akgül, 2015). Especially HPV16 expressing viral E6 and E7 oncoproteins inactivate p53 and RB tumor suppressor proteins and thus support the tumorous cell proliferation. Recently, HPV16-positivity has been routinely tested during diagnosis, and patients with HPV16 detected in the tumorous tissue are predicted to respond well to radiotherapy (de Abreu et al., 2018). Moreover, HPV-positive head and neck squamous cell carcinoma cases are less likely to develop PNI than HPV-negative cases (Zhang et al., 2019).

The association of HPV infection with other epigenetic factors has been determined in OSCC (Klussmann et al., 2009; Sartor et al., 2011; Jithesh et al., 2013). Downregulation of methylation in CCNA1 promotor was found in HPV-positive patients with OSCC (Sartor et al., 2011). On the contrary, hypermethylation was proven in CCNA1, DCC, TIMP3, EYA4, and WT1 genes in HPV-positive OSCC (Viet and Schmidt, 2008; Arantes et al., 2015). The methylation status of the HOXA9 gene, encoding a homeobox protein, could even serve as a biomarker for early detection of usually HPV-negative OSCC (Guerrero-Preston et al., 2011). We also found other robustly methylated regions, specific for HPV + OPSCC, such as KCNA3, EMBP1, CCDC181, DPP4, ITGA4, BEND4, ELMO1, SFMBT2, C1QL3, MIR129-2, NID2, HOXB4, ZNF439, ZNF93, VSTM2B, ZNF137P, and ZNF773 (Ren et al., 2018). We have demonstrated the link between the abundant DNA methylation, chromatin modifications, and gene expression for this disease (Guo et al., 2017; Ando et al., 2019; Guo et al., 2020). Moreover, our recent work demonstrated that HPV causes epigenetic deformation at the site of viral integration into the host genome (Kelley et al., 2017). Based on these observations, it was proposed that viral-related tumors have different epigenetic patterns than chemical-related tumors and healthy patients (Faraji et al., 2018).

## 7 Possible Therapeutic Targets and Recent Clinical Trials

Perineural invasion is widely regarded as an indicator of poor prognosis in oral cancer patients. It significantly correlates with aggressiveness of tumor, disease recurrence, and increased morbidity and mortality. Radiation is usually indicated for patients with head and neck SCC with PNI after resection as adjuvant therapy (Bakst et al., 2019; Bur et al., 2016). Unfortunately, dose selection, risk of catastrophic failure, and possible toxicity to nearby physiological tissues make radiation not always beneficial for patients. Therefore, focusing on the molecular basis of perineural invasion and perineural spread and epigenetic mechanisms playing a role in PNI could enable us to target nerve invasion independently from cancer itself. Such therapy would broaden treatment options for neurotropic cancers. To date, there are no clinical trials focused on PNI; therefore, we summarize epigenetic approaches used in OSCC therapy in general.

As histones are found to be highly hyperacetylated in OSCC patient samples, this implicates a HAT inhibitor as a potential therapeutic molecule. Inhibition of HAT by small molecule inhibitor Hydrazinocurcumin (CTK7A) was proven to substantially reduce the xenografted oral tumor growth in mice (Arif et al., 2010). Also, Zebularine is an inhibitor of DNA methyltransferase, which seems to be effective in OSCC therapy (Jha et al., 2015). In combination with cisplatin, Zebularine promotes cell death via an apoptotic pathway. On the other hand, the combination of Zebularine with 5-fluorouracil minimized the efficiency of Zebularine (Suzuki et al., 2009). Another DNA methyltransferase inhibiting analog, Azacitidine, in combination with Cisplatin, has been used in clinical trials for patients with advanced lung or Head and Neck Cancer and recurrent or metastatic Squamous Cell Carcinoma of the Head and Neck. Unfortunately, both clinical studies have been terminated prematurely due to study enrollment issues (ClinicalTrials.gov Identifier: NCT00901537 and NCT00443261).

Also, histone deacetylase inhibition constitutes an attractive target for the therapy of OSCC. The main benefits of HDAC inhibition consist of promoting the tumor suppressor genes activity and preserving the loose structure of chromatin. One of the most promising results has been shown in a combination of HDAC inhibitor MS-275 with chemotherapeutic agent cisplatin, which leads to the inhibition of malignant tumor behavior (Sato et al., 2006). Furthermore, the treatment of OSCC cell lines by HDAC inhibitor Entinostat resulted in reduced proliferation followed by cell cycle arrest at the G0/G1 phase and substantial apoptosis induction (Marques et al., 2020). The recent study examined the antitumor activity of Apicidin in OSCC in murine models. Apicidin inhibited cell growth through HDAC8 inhibition *in vitro* and *in vivo*, indicating that Apicidin may be a new effective therapeutic agent for OSCC (Ahn, 2018).

miRNAs therapeutic approach also has an increased potential in oral cancer administration of the epigenetic profile (Wang and Wu, 2009). The traditional perspective involves introducing exogenous tumor suppressor sequences for their enhanced expression (Saito et al., 2006). Another approach could be to restore miRNAs expression that inhibits the aberrant activity of enzymes under normal conditions, e.g., DNA methyltransferase. The constant issue of this therapeutical approach consists of the non-specificity of these epigenetic modifier drugs because healthy cells could be affected, or previously suppressed oncogenic genes in tumor cells might be activated (Irimie et al., 2018).

## 8 Conclusion

Here, we summarized the association of epigenetic regulation of genes related to squamous cell carcinoma with special attention on perineural invasion from different points of view, including alterations of DNA and histones modification up to epigenetic factors such as alcohol, smoke, or HPV-positivity and their possible effect on these tumorous cells.

While previously, attention was mainly paid to epigenetic changes in HNSCC, their relation to the perineural invasion was at the edge of the attention. As there is a close relationship between perineural invasion and tumor invasiveness, inhibition of perineural invasion through regulation of epigenetic changes could open new avenues for effective cancer treatment in these patients.
