# Supplementary material for: Epigenetic Regulations of Perineural Invasion in Head and Neck Squamous Cell Carcinoma
Source: Front Genet. 2022 Apr 27;13:848557. doi: 10.3389/fgene.2022.848557 (PMC9091179; doi:10.3389/fgene.2022.848557)
Supplement: Supplementary file 1 [file Table1.DOCX]

**Table S1:** **DNA and histone epigenetic modifications associated with PNI**

| Gene with altered methylation status | Association with PNI in OSCC/HNSCC | Association with PNI in other neurotropic cancers | (Ref.) |
| --- | --- | --- | --- |
| TFPI2 | - | Pancreatic cancer | (Zhai et al., 2015) |
| CDKN2A | - | Prostate cancer | (Verdoodt et al., 2011) |
| CDH1 | - | Salivary gland adenoid cystic carcinoma | (Zhang et al., 2007) |
| PTEN | - | Prostatic adenocarcinoma | (Kim et al., 2015) |
| HOXA1 | HNSCC |  | (Li et al., 2021) |
| RUNX3 | - | Salivary gland adenoid cystic carcinoma | (Ge et al., 2011) |
| NTRK1 | - | Pancreatic cancer | (Zhu et al., 1999) |
| GFRA1 | - | Pancreatic cancer cells; Bile duct carcinoma | (Gil et al., 2010); (Iwahashi 2002) |
| TNFRSF10C | - | Pancreatic adenocarcinoma | (Dauksa et al., 2012) |
